# Combining theranostic/particle therapy with immunotherapy for the treatment of GU malignancies

**DOI:** 10.1002/bco2.316

**Published:** 2023-12-13

**Authors:** Karin A. Skalina, Beata Małachowska, Kunal K. Sindhu, Marcher Thompson, Anthony D. Nehlsen, Lucas Resende Salgado, Zachary Dovey, Shaakir Hasan, Chandan Guha, Justin Tang

**Affiliations:** ^1^ Department of Radiation Oncology Montefiore Medical Center/Albert Einstein College of Medicine Bronx New York USA; ^2^ Department of Radiation Oncology Icahn School of Medicine at Mount Sinai New York New York USA; ^3^ Department of Radiation Oncology AIS Cancer Center/Adventist Health Bakersfield California USA; ^4^ Department of Urology Icahn School of Medicine at Mount Sinai New York New York USA; ^5^ New York Proton Center New York New York USA

**Keywords:** abscopal effect, cancers, genitourinary, immunotherapy, particle therapy, theranostics

## Abstract

Particle therapy and radiopharmaceuticals are emerging fields in the treatment of genitourinary cancers. With these novel techniques and the ever‐growing immunotherapy options, the combinations of these therapies have the potential to improve current cancer cure rates. However, the most effective sequence and combination of these therapies is unknown and is a question that is actively being explored in multiple ongoing clinical trials. Here, we review the immunological effects of particle therapy and the available radiopharmaceuticals and discuss how best to combine these therapies.

## 1 INTRODUCTION

Although radiation therapy (RT) has historically been categorized as a local therapy, the systemic or abscopal effects of RT were reported as early as the 1950s.^1^ This view of RT as a ‘systemic treatment’ has become increasingly relevant, as we understand more about the effect of RT on tumour cells and the tumour microenvironment (TME) from an immunological aspect. The TME, including T lymphocytic infiltration, intra‐tumoral myeloid activation, major histocompatibility complex (MHC) expression and tumour antigens, predicts a tumour's response to immunotherapy and its ability to evade immunological detection.^2^ A tumour that evades the immune system and is resistant to immunotherapy is typically referred to as ‘cold’, whereas one with increased immune cell infiltration that is more likely to respond to immunotherapy is referred to as ‘hot’. RT can convert a cold TME into an immunologically active tumour by recruiting anti‐tumour immune cells and shifting the cytokine balance.^3^ More recent evidence has demonstrated the type of RT can also impart different immunological consequences. As oncological centres investigate combination therapies, the understanding of radiation dose as energy deposited may need to be redefined in the context of theranostic and particle therapy to gain more insight into its impact as immunotherapy moves to frontline therapy in cancer care.^4^ This article reviews the current evidence and potential applications for combined theranostic/particle therapy with immunotherapy and how modulating the dose and type of RT delivery can synergize the systemic effects of modern immunotherapy.

## 2 IMMUNE ACTIVATION WITH PHOTON RADIATION THERAPY

RT directly kills tumour cells by inducing DNA strand breaks, which elicit a stress response, including DNA damage response, the unfolded protein response and autophagy. In cases where the damage is irreparable, malignant cells will either enter senescence or undergo regulated cell death.^5^ The radiation‐induced damage is directly related to the dose delivered. In contrast, the immunologic response to radiation, which can be immune‐stimulatory or inhibitory and results in indirect tumour cell death, is not dependent on dose delivered.^5^, ^6^, ^7^, ^8^ Radiation causes antigen release for immune presentation during cell death^9^ and increasing expression of MHC I proteins on both tumour^10^, ^11^ and immune cells. The release of damage‐associated molecular patterns (DAMPs) and pro‐inflammatory cytokines after radiation recruits antigen‐presenting cells and T cells into the tumour.^12^, ^13^, ^14^ In some cases, cells undergo immunogenic cell death (ICD) wherein calreticulin is exposed on the cell surface, adenosine triphosphate is secreted, and high mobility group box 1 (HMGB1) protein is released, triggering an immune cascade resulting in cross‐presentation to cytotoxic T lymphocytes.^12^, ^14^, ^15^, ^16^, ^17^ There are many studies analysing the photon radiation dose and fractionation dependency of ICD.^18^ In recent years, the potential of RT to enhance systemic anti‐tumour immunity has gained considerable attention. Photon radiation has been shown to promote ICD (as well as non‐immunogenic or tolerogenic cell death) in addition to immunogenic modulation in surviving cells. These effects may be synergistic with immunotherapeutic regimens.^19^

## 3 IMMUNE ACTIVATION WITH PARTICLE THERAPY

Particle therapy utilizing protons and heavier ions (i.e., carbon ions) is becoming more available in the United States and throughout the world. Both protons and carbon ions have higher linear energy transfer (LET) and increased relative biological effectiveness (RBE) than traditional photon therapy. Because of the relative novelty of these therapies, the exact immune effects have not been fully determined. It is well known, however, that particle therapy can improve normal tissue sparing and more efficiently kill tumour cells.^20^, ^21^, ^22^ An in vitro study was conducted to determine if photon, proton and carbon ion therapy have similar effects on the first step in ICD, the cell‐surface translocation of calreticulin. Although all three therapeutic types exhibited a time‐dependent increase in calreticulin exposure, proton and photon therapy had a dose‐dependent effect, whereas carbon ion did not.^23^ Yoshimoto et al. determined both photon and carbon ion therapy significantly increased HMGB1 in the supernatant of human cell cultures to similar amounts.^24^ In a preclinical study of cell lines, both proton and photon radiation induced similar immunogenic signal upregulation (e.g., calreticulin and tumour‐associated antigens) as well as similar antigen‐specific cytotoxic T lymphocyte killing.^25^

Mirjolet et al. investigated the immune response in murine colon cancer after a single dose of proton beam RT. Seven days post‐treatment, increased tumour infiltration of anti‐tumour and pro‐tumour immune cells was observed. CD8+ T cells also expressed more granzyme B, an enzyme necessary for direct cytotoxicity, after treatment with proton beam radiation compared with control.^26^ In a preclinical study of murine breast cancer, unlike photon therapy, low‐dose (≤4Gy) carbon ion therapy did not result in cytotoxic (CD8+) T lymphocyte cell death.^27^ Additionally, 4Gy carbon ion radiation, not photon, increased production of granzyme B, interleukin 2 and tumour necrosis factor α by tumour‐infiltrating CD8+ T cells.^27^ Similar results were seen in 32 patients undergoing carbon ion therapy for prostate cancer whereby circulating CD4+ and CD8+ lymphocytes, as well as natural killer cells, were elevated after carbon ion therapy but not after proton therapy. The production of pro‐inflammatory cytokines and the proliferation of these immune cells was also increased following carbon ion therapy.^28^ The influx of pro‐inflammatory immune cells often accompanies immunosuppressive cells, such as myeloid‐derived suppressor cells (MDSCs), which inhibit T cell function and enhance the proliferation and differentiation of malignant cells. In murine melanoma, tumour irradiation with carbon ions decreased the number of circulating, splenic and intratumoural MDSCs when compared with photon radiation or no treatment. Additionally, regulatory T cells were decreased and CD8+ T cells increased in circulation and in the tumour with carbon ion when compared with X‐rays, improving survival in mice treated.^29^ Although the mechanism behind the lymphocyte‐sparing and pro‐inflammatory effects of carbon ions has not been fully elucidated, the potential for induction of a potent anti‐tumour immune response is promising.

In a study comparing chromosomal aberrations in peripheral blood lymphocytes of prostate cancer patients undergoing carbon ion or photon beam radiation treatment, less aberrations were seen in patients treated with carbon ions compared with photon therapy.^30^ Previous studies in oesophageal and uterine cervical cancer patients also demonstrated reduced cytogenic damage in peripheral lymphocytes in patients treated with carbon ions compared with those treated with photon beam. The cytogenetic damage was a function of field size with photon therapy but not with carbon ion.^22^ These studies indicate that there may be an inherent resistance to particle therapy in immune cells. Proton therapy's normal tissue sparing has significantly reduced treatment‐associated lymphopenia in bladder cancer patients.^31^ Maintaining adequate circulating lymphocytes is critical to promoting an immune response and harnessing the full potential of immunotherapeutic agents.

**FIGURE 1:**
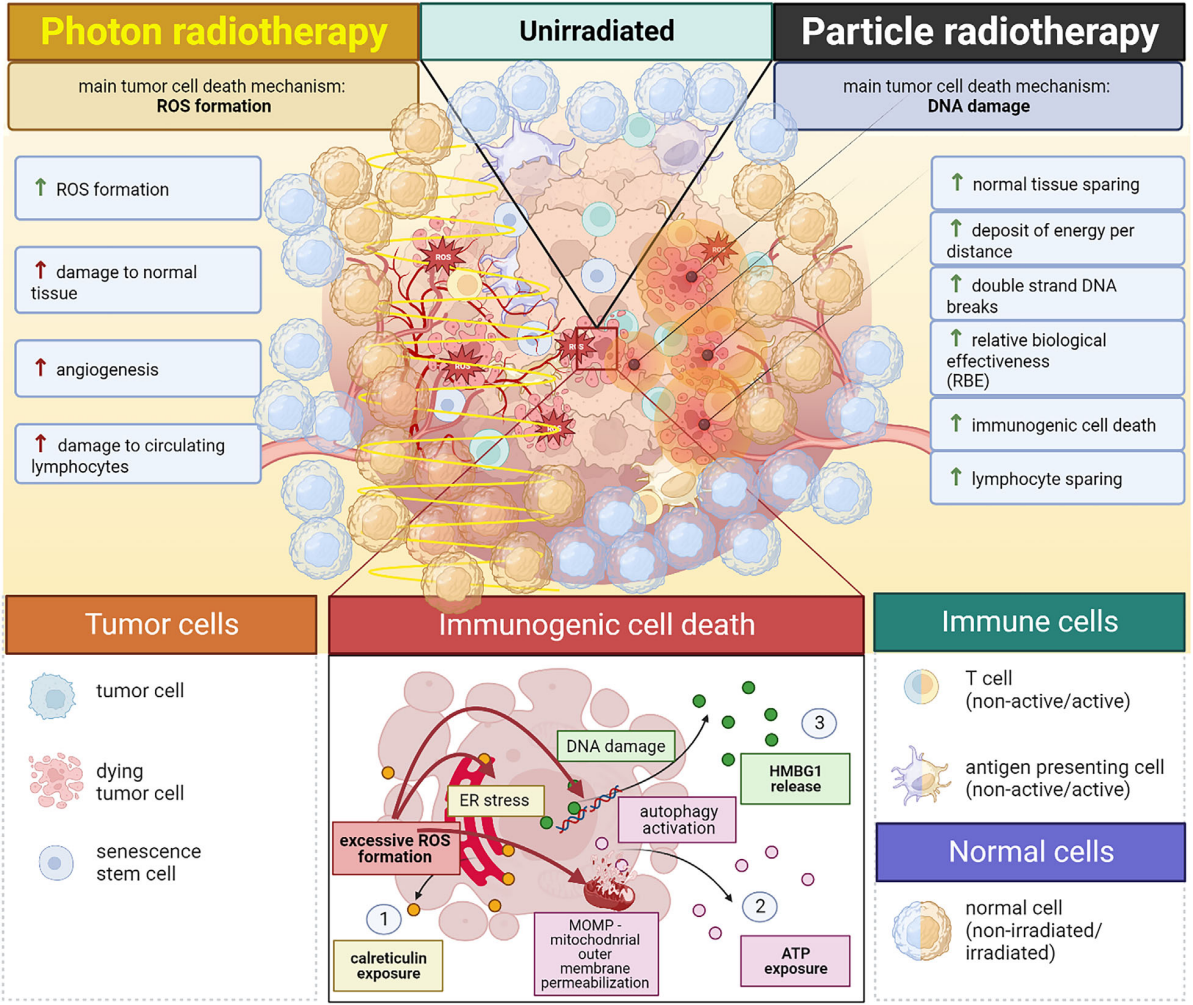
Summary of effects of photon versus particle therapy cancer and immune cells.

It is important to note that the mechanisms of immune activation with particle therapy are still under active investigation, and further research is needed to fully understand the complex interactions between radiation and the immune system. Particles with their higher LET deposit more energy per distance, producing more double‐stranded DNA breaks and possibly immune system activation.^3^ Therefore, because of efficient tumour cell death and immune cell sparing, particle therapy may be more synergistic with immunotherapeutic than photon therapy. The immunological differences of particle and photon therapy are summarized in Figure 1.

## 4 RENAL CELL CARCINOMA

Renal cell carcinoma (RCC) accounts for only 2% of annual global cancer diagnoses.^32^ The 5‐year survival rate for localized RCC is about 95% but drops to less than 20% for metastatic disease.^33^ Although surgery is the first‐line treatment for patients with localized disease, stereotactic body radiation therapy (SBRT) can be an alternative definitive therapy in patients who are poor surgical candidates. In a prospective study of 74 patients with localized, unresectable RCC treated with SBRT, the cumulative incidence of local failure was 7.77% at 4 years with an optimal dose of 40Gy in 5 fractions.^34^ Several recent studies have demonstrated that SBRT in the oligometastatic setting can prolong survival and delay disease progression,^35^, ^36^, ^37^ including RCC.^38^ RCC is traditionally considered to be chemotherapy resistant, and first‐line therapy for metastatic disease involves immune checkpoint blockade (ICB) and targeted therapy with tyrosine kinase inhibitors in addition to cytoreductive surgery or RT. Unlike surgery, RT, as discussed, can promote antigen release and induce anti‐tumour immunity. The NIVES study investigated the use of RT and ICB in patients with metastatic RCC who progressed after prior anti‐angiogenic therapy with at least two nonbrain sites of disease. Patients were treated with nivolumab and underwent SBRT to one disease site after initiation of nivolumab. Although only 12 patients (17%) achieved partial or complete response, the objective response rate was 29% in the irradiated lesion versus 12% in the nonirradiated lesion, indicating that the combination therapy is more beneficial than immunotherapy alone.^39^

## 5 BLADDER CANCER

Bladder cancer was one of the first cancers successfully treated with a form of immunotherapy after minimally invasive surgical resection. Bacillus Calmette Guerin (BCG) is a live‐attenuated version of *Mycobacterium bovis* initially used as a vaccine for tuberculosis in the early 20th century.^40^ BCG was first established as a therapy for non‐muscle invasive bladder cancer more than 40 years ago and maximal efficacy requires an immunocompetent patient, live BCG and intravesical (local) instillation. Although its exact mechanism of action is still not fully understood, it is thought to impart direct cytotoxicity and indirect cytotoxicity through immune activation.^41^ For muscle‐invasive bladder cancer (MIBC), radical cystectomy (RC) remains the standard of care (SoC) but there is an increasing body of evidence for the use of bladder sparing approaches, combining minimally invasive surgical resection with chemotherapy, radiotherapy and immunotherapy, especially in patients not fit for RC or those wanting to avoid urinary diversion. Two multicentre, phase III trials, BC2001 and BCON, established that hypofractionated RT in combination with chemotherapy or hypoxia modification can be an alternative to cystectomy.^42^, ^43^ Similarly, tri‐modality therapy (TMT) [defined as transurethral resection of bladder tumour (TURBT), radiation and chemotherapy] has been investigated although not by prospective randomized study. A meta‐analysis reviewing six bladder preservation studies demonstrated 5‐year overall survival (OS) and cancer‐specific survival (CSS) of approximately 50% and 70%, respectively, with 21% of patients requiring salvage cystectomy.^44^ A number of other observational studies have compared oncological outcomes between RC and TMT, demonstrating 5‐year OS and CSS rates of 50% and 84% for RC, compared with 36% and 74% for TMT.^45^ The emphasis through all bladder sparing studies is the importance of patient selection ensuring patients are without invasion beyond the muscularis propria, hydronephrosis, carcinoma in situ or multifocal disease.^46^ Nevertheless, both TMT and the trials discussed above (BC2001 and BCON) are associated with 10‐year OS of only 30%, with increasing salvage cystectomy rates.

After non‐small cell lung cancer and melanoma, bladder cancer has the highest prevalence of mutations, correlating with high immunogenicity^47^ and perhaps responsiveness to ICB. In a small study, the monoclonal antibody to EGFR, cetuximab, was added to TMT regimen from BC2001 to determine its safety and effect on local recurrence rate. Although hampered by small population and power, 93% of patients did not have muscle invasion at 1 year compared with the 82% in BC2001,^48^ indicating that immunotherapy can improve outcomes. Another small, single‐arm phase II trial (DUART) investigated the combination of durvalumab and RT followed by maintenance durvalumab for patients with localized MIBC who were ineligible for cisplatin or surgical resection. Combination therapy in these 26 patients was safe with minimal toxicity (no grade 3 or higher radiation cystitis or proctitis) and comparable results to other trials with an 83% overall response rate at 1 year.^49^ Two ongoing large, randomized clinical trials (Keynote992 and RadIO) are investigating the addition of ICB to TMT for MIBC.^50^ Additionally, a new Swiss clinical trial SAKK 06/19 is investigating intravesical recombinant BCG and intravenous atezolizumab followed by neoadjuvant cisplatin/gemcitabine and then RC and pelvic lymphadenectomy with maintenance atezolizumab in MIBC on pathologic complete response.^51^

In metastatic cancers, ICB monotherapy is often utilized. A small, randomized phase I trial with 18 total patients investigated the timing of SBRT (24Gy in 3 fractions) in combination with pembrolizumab for patients with metastatic urothelial carcinoma. SBRT was administered either prior to 1st cycle of pembrolizumab or 3rd cycle of pembrolizumab. In the nine patients who received SBRT first, a partial response was seen initially followed by progression of disease. However, in the nine patients receiving SBRT in between pembrolizumab cycles, four had sustained response to the combination therapy,^52^ indicating that the immune system's checkpoints need to be suppressed before antigens are released by RT. These results led to a larger, phase II trial of 96 patients, the Checkpoint Inhibition in Combination with an Immunoboost of External Beam Radiotherapy in Solid Tumours (CHEERS), which randomized patients with metastatic cancer of varying histologies, including RCC and urothelial carcinoma, to receive SBRT (8Gy ×3) after 2 cycles of ICB. Recently published results did not demonstrate improved progression‐free survival (PFS) or OS with the addition of SBRT.^53^ It should be noted that this SBRT dose, however, was optimized to improve immunogenicity of the tumour and not provide efficient cell killing, both of which are needed to increase survival.

## 6 RADIOPHARMACEUTICAL THERAPY IN PROSTATE CANCER

Radiopharmaceutical therapy is the delivery of radioactive atoms with tumour‐associated targets. Unlike external beam radiation that delivers the same absorbed dose regardless of the number of tumour cells, the absorbed dose of a radiopharmaceutical agent is dependent on the number of cells clustered together. The larger the cluster of tumour cells, the greater the absorbed dose.

### 6.1 Radium‐223

More than 90% of patients with metastatic castration‐resistant prostate cancer (mCRPC) have bone metastases which negatively impacts quality of life and whose complications can lead to death. Systemic therapy with bone‐seeking particles can provide symptomatic relief. Αlpha‐emitters are relatively heavy particles that produce densely ionizing tracks through tissue, have a short range and cause mostly irreparable double‐stranded DNA breaks.^54^ Within bone, the short range of α‐particles allows for efficient energy delivery to the bone surface, while sparing the bone marrow, reducing myelosuppression.^55^ Radium‐223 is a cationic, natural bone‐seeking molecule, which has been studied in the treatment of painful bone metastases.^56^ The Alpharadin in Symptomatic Prostate Cancer Patients (ALSYMPCA) was a double‐blind, randomized, international, phase III clinical trial comparing Radium‐223 to placebo for the treatment of patients with progressive mCRPC and more than two bony metastases. The use of radium‐223 resulted in a 30% reduction in risk of death as well as an improvement in quality of life.^57^, ^58^ Combination of radium‐223, however, with the androgen suppressant abiraterone and prednisone did not improve symptomatic skeletal event‐free survival and resulted in more bone fractures.^59^ Combinations with other androgen‐deprivation therapies that do not cause mineralocorticoid excess may prove more efficacious.

### 6.2 Brachytherapy

Brachytherapy is a form of RT, involving the placement of radioactive sources with the short range of penetration. Studies vary greatly on the dose of radiation needed to promote immunogenicity of a tumour^9^, ^60^, ^61^ as well as the tissue sparing that must occur in order to preserve active immune cells. With brachytherapy, the placement of an internal radioactive source creates a dose cloud based on the distance from the source, resulting in a heterogeneous tissue response where tissues closest to the source undergo cell death and tissues farther away release immunostimulatory cytokines and have increased immune cell infiltration.^62^ Several preclinical studies have been performed brachytherapy that can act synergistically with ICBs, resulting in an abscopal effect.^63^

Diffusing alpha‐emitter Radiation Therapy (DaRT) is a specific type of interstitial brachytherapy, utilizing radium‐224, an alpha‐emitting radionuclide, capable of effectively destroying numerous types of tumours, including prostate cancer, in preclinical models.^64^, ^65^, ^66^ The distribution of alpha particles in the tumour depends on the tumour tissue density, blood vessel availability and the sensitivity of the tumour to radiation. In mouse models of ‘hot’ colon carcinoma and ‘cold’ breast carcinoma, treatment of the primary tumour with DaRT generated anti‐tumour immunity that delayed the growth of a ‘rechallenge’ tumour, reduced the metastatic burden and prolonged survival.^67^ Combination of DaRT with toll‐like‐receptor (TLR) agonists and regulatory T cells and MDSC inhibitors enhanced tumour control and anti‐tumour immunity in a mouse model of colon carcinoma.^68^ DaRT has also been tested with anti‐programmed death 1 (PD1) therapy in murine squamous cell carcinoma, demonstrating synergy with regard to anti‐tumour immune activation and tumour control.^69^ In the clinical setting, DaRT to a single cutaneous squamous cell carcinoma lesion in a patient with multiple synchronous lesions resulted in regression of the treated lesion as well as distant, untreated lesions, indicating that the immune activation observed in preclinical models could be applicable to patients.^70^

DaRT has received Breakthrough Device Designation by the United States Federal Drug Administration for the treatment of squamous cell carcinoma of the skin or oral cavity without curative SoC and for recurrent glioblastoma. A summary of open clinical trials investigating radiopharmaceuticals for genitourinary cancers is in Table 1.

**TABLE 1:** Selection of active clinical trials in genitourinary cancers utilizing particle therapy.

Trial name	Trial location	Cancer type	Modality	Primary outcome(s)	Planned # Pts	Status	Projected completion date
NCT04543903	Israel	Prostate cancer planned for prostatectomy	DaRT	Feasibility & AE	10	Recruiting	05/2024
NCT04576871	United States	Progressive mCRPC	225Ac‐J591	Safety	18	Recruiting	12/2024
NCT05567770	United States	mHSPC	225Ac‐J591 & SBRT	DLT & change in MTD	24	Not Yet Recruiting	01/2030
NCT04886986	United States	Progressive mCRPC	225Ac‐J591 ± 177Lu‐PSMA	DLT, MTD and PSA decline	33	Recruiting	12/2027
NCT04597411	Australia & South Africa	PSMA‐positive prostate cancer	225Ac‐PSMA‐61	Recommended phase 2 dose	60	Recruiting	07/2025
NCT04644770	United States	mCRPC	225Ac‐labelled anti‐human kallikrein‐2	DLT & AE	100	Recruiting	07/2025
NCT04946370	United States	Progressive mCRPC	ICB + ARPI ± 225Ac‐J591	DLT, optimal dose of 225Ac‐J591 for phase II & response rate	76	Recruiting	06/2028
NCT05204927	United States, Spain, France, Italy	mCRPC	177Lu‐PSMA vs. ARPI	Radiographic PFS	400	Recruiting	06/2029
NCT05849298		PSMA‐positive CRPC	177Lu‐PSMA ± ARPI	PSA response	120	Not Yet Recruiting	01/2029
NCT05162573	Netherlands	Node‐positive prostate cancer	Concurrent EBRT + 177Lu‐PSMA	MTD	18	Recruiting	12/2023
NCT04720157	United States, Europe, Canada, Japan, China, Singapore, Korea	Metastatic HSPC	SoC ± 177Lu‐PSMA	rPFS	1126	Recruiting	02/2026
NCT03737370	United States	mCRPC	Docetaxel + radium‐223	Incidence of DLT	25	Recruiting	12/2024
NCT05133440	United States	Oligometastatic prostate cancer	SBRT ± 223Ra	PFS	136	Recruiting	11/2027
NCT03317392	United States	Metastatic prostate cancer	Olaparib + 223Ra	MTD of Olaparib and 223Ra & radiographic PFS	133	Recruiting	11/2023
NCT04071223	United States	Metastatic renal cell carcinoma with bone metastases	Cabozantinib ± 223Ra	Symptomatic skeletal event‐free survival	210	Recruiting	10/2024
NCT03361735	United States	Oligometastatic HSPC	ADT + SBRT + 223Ra	Time to treatment failure & objective response rate	24	Recruiting	12/2023
NCT04071236	United States	mCRPC	223Ra ± peposertib ± avelumab	DLT & rPFS	90	Recruiting	01/2024
NCT05893381		Oligometastatic prostate cancer	SBRT ± 177Lu‐PSMA	12‐month PSA PFS	70	Not Yet Recruiting	04/2028
NCT05150236	Australia & New Zealand	mCRPC	177Lu‐PSMA ± ICB	1‐year PSA PFS	110	Recruiting	12/2024
NCT04443062	Cyprus & Netherlands	Oligometastatic prostate cancer	177Lu‐PSMA vs. SoC	Progression and time to progression	58	Recruiting	01/2025

Abbreviations: ARPI, androgen receptor pathway inhibitor; DLT, dose‐limiting toxicity; MTD, maximum tolerated dose; AE, adverse events; SoC, standard of care; mCRPC, metastatic castration‐resistant prostate cancer; HSPC, hormone‐sensitive prostate cancer.

### 6.3 PSMA‐targeted therapy

Targeted radionuclide therapy involves the systemic administration of a radiolabelled drug. Small molecules or antibodies targeting a molecular alteration expressed by malignant cells are labelled with a β‐ or α‐emitting radioisotope. Pre‐treatment imaging with the same small molecule labelled with a positron or γ‐emitting radionuclide is used to visualize the distribution of the target.

Prostate‐specific membrane antigen (PSMA) is a type II transmembrane glycoprotein with a large extracellular portion. Despite its name, PSMA is also expressed on other tissues including salivary glands, duodenal mucosa, RCC and colon carcinoma^71^, ^72^ albeit 100–1000 fold less than on prostate epithelium.^73^ Its overexpression on prostate tumour tissue is positively correlated with Gleason score, metastatic status and hormone resistance.^74^, ^75^ There is no known natural ligand for PSMA, and the mechanism behind upregulation in advanced prostate cancer is not known.^76^ PSMA ligands can be developed with a high binding affinity to PSMA and efficient internalization into prostate cancer cells. A larger alternative to a small molecule ligand is a monoclonal antibody. The anti‐PSMA monoclonal antibody, J591, has been studied extensively linked to both α‐ and β‐emitting isotopes for therapeutic applications (Table 1). J591 binds to a different extracellular domain of PSMA compared with PSMA ligands, allowing for simultaneous use.^77^, ^78^ Monoclonal antibodies are more specific and have less off‐target effects than ligands, which can potentially reduce adverse events. PSMA ligands or antibodies can be labelled with different molecules, such as Gallium‐68 (^68^Ga) for positron emission tomography (PET), or yttrium‐90 or Lutetium‐177 (^177^Lu), for therapies.^79^ Used in combination, ^68^Ga‐PSMA‐11 can identify patients who may respond to PSMA‐directed therapy. Lutetium‐177 delivers beta‐particulate emission with a 0.7‐mm mean path length, minimizing normal tissue toxicity. Published in February 2021, TheraP, the first randomized clinical trial, utilizing a radiolabelled PSMA ligand, ^177^Lu‐PSMA‐617, compared ^177^Lu‐PSMA‐617 to cabazitaxel in men with progressive mCRPC. Men treated with ^177^Lu‐PSMA‐617 had delayed disease progression and significantly improved progression‐free survival at 12 months (19% vs. 3%).^80^ Later the same year, the results of the phase III international clinical trial, VISION, were published, which examined the addition of a ^177^Lu‐PSMA‐617 to SoC in men with mCRPC and at least one PSMA‐positive metastatic site. SoC in this trial allowed hormonal therapy and RT, but not other cytotoxic agents. The addition of ^177^Lu‐PSMA‐617 significantly improved imaging‐based PFS and OS when compared with SoC.^81^ In March 2022, the FDA approved ^177^Lu‐PSMA‐617, also known as Pluvicto™, for patients with mCRPC who have previously received chemotherapy.^82^

**FIGURE 2:**
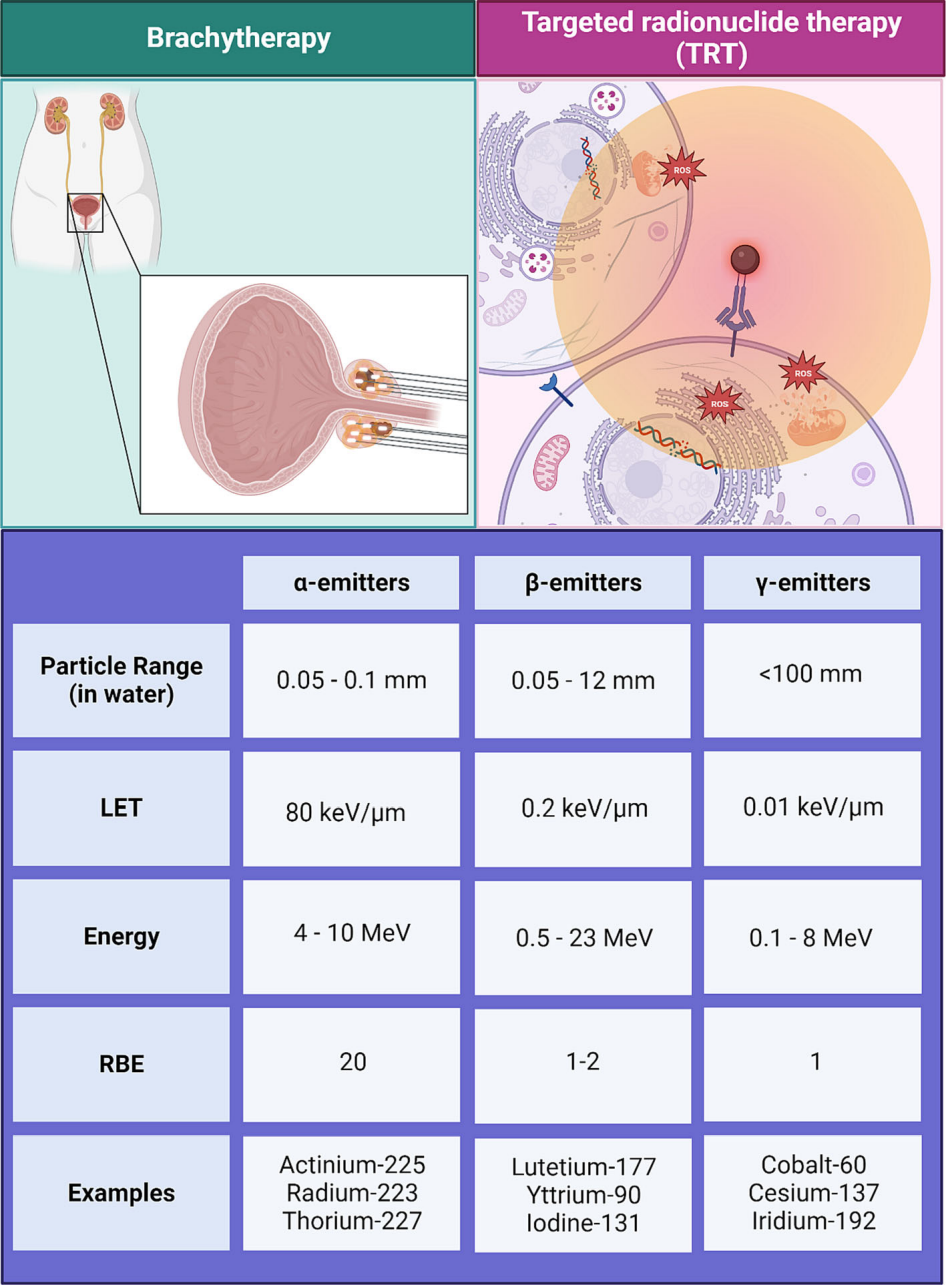
Summary of brachytherapy and targeted nuclide therapeutic physical features.

Compared with β‐emitting isotopes, α‐emitting radioisotopes (such as Actinium‐225/^225^Ac) have higher energy deposition and LET (shorter particle range), leading to reduced repair of double‐stranded DNA breaks and efficacy in hypoxia tumours (Figure 2).^83^ In a study of 40 patients with mCRPC, treatment with ^225^Ac‐PSMA improved OS and PFS, but four patients discontinued therapy because of intolerable xerostomia and 15 patients refused more treatment because of severe xerostomia.^84^ To reduce toxicity of ^225^Ac and resistance to ^177^Lu, efforts are underway to combine both therapies. In a small study of 20 patients who had an insufficient response to ^177^Lu‐PSMA‐617 monotherapy, a single treatment of ^225^Ac‐PSMA‐617/^177^Lu‐PSMA‐617 tandem therapy was administered. About 80% of patients had a PSA decline after therapy, and 50% of patients had a partial response to therapy when evaluated 6–8 weeks after infusion. Thirteen patients reported xerostomia grade 2 or lower.^85^ An alternative therapy is thorium‐227, another α‐emitter that immediately decays to radium‐223. When conjugated to a human PSMA‐targeting antibody, thorium‐227 is an attractive therapy for patients who have failed β‐emitting therapy and is currently undergoing further investigation.^86^ It is not yet known whether thorium‐227 conjugated therapy will also be limited by xerostomia.

## 7 COMBINATION THERAPIES

Although many trials, including the most recent KEYLYNK‐010, have investigated the use of ICBs alone or in combination, the immunosuppressive microenvironment in prostate cancer has prevented their success.^87^, ^88^, ^89^, ^90^ Updated results of the phase III randomized trial PEACE‐1 were presented at ASCO2023 demonstrating that in men with de novo, metastatic castration‐sensitive prostate cancer, the addition of prostate RT to SoC and abiraterone/prednisone improved radiographic PFS and OS. RT and SoC only did not have improved outcomes compared to SoC alone.^91^ These results add to the growing body of literature establishing that RT to the prostate^92^, ^93^ or to metastatic sites^36^, ^94^, ^95^ improve outcomes in de novo metastatic prostate cancer patients. Although the advantage of a brachytherapy boost has been proven in localized high‐risk patients,^96^ de novo oligometastatic patients could benefit as well. The dose heterogeneity provides ablation of a large burden of disease as well as immune stimulation and priming to promote systemic activity and prolong PFS. In patients who are not brachytherapy candidates, the use of spatially fractionated RT or particle therapy could also provide similar immune stimulation and immune cell sparing, with subsequent improvement in outcomes. With the advent of radiopharmaceuticals, the addition of primary tumour therapy to systemic therapy may provide significant benefit for metastatic patients as well.

To mount an effective anti‐tumour immune response, the following steps must take place: (1) tumour‐associated antigen recognition and presentation, (2) immune activation and proliferation and (3) tumour cell detection and death. These steps require immune cells access not only to dead or dying tumour cells but also to the live tumour cells. Dendritic cells must reach the tumour first in order to generate chemokines, such as CXCL9 and CXCL10, which recruit effector T cells to the tumour, resulting in tumour control.^97^ Thus, when planning combination therapies with immunotherapy, consideration of the timing of different types of therapy is crucial. As discussed above, both proton and carbon ion therapy spare immune cells more than photon radiation, which could provide better synergy with immunotherapy, as the presentation of tumour‐specific antigens is not hampered by death of immune cells in close proximity. Delivery of systemic therapies, such as radiopharmaceuticals and immunotherapy, is limited by circulation and thus may be hampered in larger tumours, which often outgrow their blood supply. Therefore, combination of therapeutics with an ablative local therapy in addition to a systemic one may be needed to provide overall control. A proposed therapeutic strategy for newly diagnosed metastatic cancer patients could be ICB, ablative therapy, followed by systemic radiopharmaceuticals. Results of clinical trials indicate that ICB prior to ablative therapy may provide more efficient immune activation. When followed by particle‐ablative therapy, the immune‐sparing effects and antigen release would further enhance the immune cell activation initiated by ICB. Lastly, a radiopharmaceutical agent, such as radium‐223 if bone metastases are prominent or a PSMA‐targeted agent if soft tissue metastases are dominant, would continue the cascade of tumour antigen release and with ICB could promote long‐lasting tumour immunity.

## 8 CONCLUSIONS

In genitourinary cancers, immunotherapy has been ineffective for prostate cancer and yet highly efficacious for RCC and bladder carcinoma. On the other hand, radiopharmaceuticals have a demonstrable role in prostate cancer treatment, but not in other GU cancers. With the expanding arsenal of radiopharmaceuticals and particle therapy, combination therapy has the potential to provide local, systemic and long‐lasting tumour control for GU cancers.

## AUTHOR CONTRIBUTIONS

Karin A. Skalina wrote the manuscript with contributions from Kunal K. Sindhu, Marcher Thompson, Anthony D. Nehlsen, Lucas Resende Salgado, Zachary Dovey, Shaakir Hasan, Chandan Guha and Justin Tang. Beata Malachowska created the figures.

## CONFLICT OF INTEREST STATEMENT

Dr Zach Dovey is the Medical Director and stock owner (with certificate of shares) of Medtech Holdings Ltd. No other authors have any conflicts to declare.
